# Conservative treatment of invasive bladder cancer

**DOI:** 10.3747/co.v16i4.411

**Published:** 2009-08

**Authors:** N.J. Rene, F.B. Cury, L. Souhami

**Affiliations:** * Department of Radiation Oncology, McGill University Health Centre, Montreal, QC

**Keywords:** Bladder cancer, conservative treatment, radiation therapy, chemotherapy, radiochemotherapy, trimodality therapy, bladder preservation

## Abstract

The concept of organ-preserving therapies is a trend in modern oncology, and several tumour types are now treated in this fashion. Trimodality therapy consisting of as thorough a transurethral resection of the bladder tumour as is judged safe, followed by concomitant chemoradiation therapy, is emerging as an attractive alternative for bladder preservation in selected patients with muscle-invasive bladder cancer. Long-term data from multiple institutional and cooperative group studies have shown that this approach is safe and effective and that it provides patients with the opportunity to maintain an intact and functional bladder with a survival rate similar to that for modern radical cystectomy.

## 1. INTRODUCTION

Urinary bladder cancer is the ninth most frequent cancer worldwide, accounting for 2.1% of all cancer deaths 1. Most of these cancers (65%–70%) are superficial at presentation and are typically managed conservatively. Bladder tumours are staged according to the depth of invasion. The main distinction, in terms of treatment and prognosis, is whether the disease invades the muscular layer of the bladder wall. In general, non-infiltrating tumours are treated with transurethral resection of the bladder tumour (turbt) plus intravesical instilled treatments (bacille Calmette–Guérin or chemotherapy); infiltrating tumours are treated in a more aggressive way.

The most commonly accepted standard local treatment for muscle-infiltrating tumours remains radical cystectomy with pelvic lymph node dissection 2. Since the late 1980s, several centers have used bladder-preserving strategies, combining radiotherapy (rt) and chemotherapy as an alternative to radical cystectomy 3–9.

In the present article, we review the most relevant literature on radiochemotherapy treatments for infiltrative transitional-cell bladder carcinoma, and we show evidence that bladder preservation is a safe approach—as effective as the current “gold standard”—that should be considered as an attractive therapeutic option for selected patients.

## 2. METHODS

We undertook a comprehensive search of the medline and PubMed databases using the descriptors “bladder cancer,” “radiotherapy,” “bladder conservation,” “chemoradiation,” and “chemotherapy.” Only papers in the English language from large reported series that included patients who were deemed fit for surgery were reviewed. Papers reporting on patients who did not systematically undergo turbt followed by chemoradiation 10–12, on a relatively small number of patients 13–16, or on patients who received unusual chemotherapy regimens (protracted venous 17 or intra-arterial 18–27 infusion) were not included in the review.

## 3. RESULTS

### 3.1 Cystectomy

Radical cystectomy is considered by most urologists to be the “gold standard” treatment for muscle-invasive transitional-cell carcinoma of the bladder. This surgery consists of the cystectomy itself, often accompanied by a prostatectomy (in some patients a prostate-sparing technique is possible) or anexohys-terectomy, lymphadenectomy, and urinary diversion. Options for urinary diversion include ileal conduit, continent cutaneous reservoir, and orthotopic neobladder 2,28,29. An orthotopic neobladder consists in reconnecting the ureters to an intestinal segment that is connected to the urethra. This procedure avoids the stoma in the abdominal wall required with the ileal conduit and the continent cutaneous reservoir.

The use in recent decades of orthotopic neobladder has encouraged most urologic surgeons to advocate cystectomy as the preferred treatment. However, not all patients are candidates for such a procedure. In a large series from the University of Southern California, Stein *et al.* 2 reported that, of 1054 surgical patients between 1971 and 1987, only 38% had an orthotopic diversion performed. The series included many patients treated before the neobladder procedure was available. If patients treated only after 1987 (when neobladder became the standard for selected men) are considered, 63% underwent the procedure, and if the analysis is further limited to patients treated after 1992, when neobladder became the standard for women as well, then 69% of patients received a neobladder.

In a urinary diversion consensus meeting convened by the World Health Organization and the Société Internationale d’Urologie 30, pooled data from 8 centers involving 7129 patients showed that only 47% had undergone an orthotopic diversion. Excluding the two series that included patients treated before 1986, a total of 53% of patients underwent orthotopic urinary diversion. Thus, not all patients are eligible for orthotopic urinary diversion, limiting the number of patients that will keep the ability to void per urethra. The inability to obtain a negative margin in the urethra in a male patient is an absolute contraindication for the procedure; relative contraindications include an incapacity to perform self-catheterization if the need arises, renal insufficiency, and hepatic dysfunction 28.

The report from the University of Southern California is probably one of the largest published radical cystectomy series in patients harbouring bladder cancer 2. Their results should be considered the “gold standard” against which other treatments should be compared. This large series of 1054 patients excluded 112 patients with inoperable disease or those who, after the surgery, were found to have positive macroscopic (gross) margins. In that series, which includes 39% pT1 or lesser tumours, the recurrence-free survival rates at 5 and 10 years were 68% and 66% respectively, and the 5- and 10-year overall survival rates were 60% and 43% respectively. Although not stated by the authors, the calculation of the disease-free survival rates may have censored patient deaths from other causes. Such censoring would explain the disease-free survival rate being higher than the overall survival rate. The local recurrence rate was 9%, and in 23% of the patients, positive lymph nodes were found at the time of the operation. The surgical mortality rate was 3%.

### 3.2 Bladder Preservation Therapy

Interest has been growing in a more conservative, organ-preserving approach to many malignant tumours. The association of a limited surgery and radiotherapy (rt) is the basis of most of the organ-preservation protocols. Today, conservative treatment for breast, larynx, esophagus, anus, and lower rectum carcinomas and limb sarcomas is the standard of care in many oncologic centers 31.

Historically, bladder-conservative treatments, mostly with rt alone 32–38, were initially used in patients who were not candidates for surgery. Despite the poorly selected population, the 5-year local control rate for these patients ranged between 35% and 45% 32,34,36, with a 5-year overall survival of 25%–40% 33–35,37–39. Those results were considered inferior to the results for surgery, and therefore rt as a single-modality therapy was rarely fully explored in a better-selected cohort of patients. A possible exception is the series from the Princess Margaret Hospital 3 in which, of 340 patients treated radically with rt, 131 had T2 disease without nodal involvement. Of those patients, 108 received rt alone. The 5-year cause-specific survival without cystectomy for all T2 patients (including those treated using a combined approach) was 52%. The use of rt alone as a first-line primary treatment has practically been abandoned.

Three randomized trials 40–42 have compared preoperative rt followed by surgery against radical rt with cystectomy reserved for recurrent local disease. Only the smallest of these studies, with 67 patients enrolled, showed a survival benefit for immediate surgery 41. The other two trials did not find a statistically significant difference in overall survival between arms at 5 years. Two population-based studies 43,44 also show that the survival rate is the same whether cystectomy is the initial treatment or whether it is reserved for possible recurrence after radical rt.

### 3.3 Trimodality Treatment: TURBT + RT + Chemotherapy

Since the late 1980s, several centers have pioneered the bladder-preserving strategy as an alternative to radical cystectomy 3,5–9,45–47. This strategy is multidisciplinary. It includes as thorough as is possibly safe turbt, followed by rt combined with chemotherapy, targeting patients with nonmetastatic muscle-invasive bladder cancer. Among several institutions using this approach, three have the largest experience, and their treatment methods and results are reviewed here. The Radiation Therapy Oncology Group (rtog) has also actively participated in the development of novel conservative treatments in the multi-institutional setting, and its experience is also reviewed.

The University of Erlangen 7, the Massachusetts General Hospital (mgh) 9, and the University of Paris 5 developed protocols that are based on similar therapeutic approaches. According to their studies, a complete response to an initial treatment consisting of turbt followed by irradiation plus chemotherapy selects patients whose tumour is likely to be controlled by a bladder-sparing approach. After several weeks of radiochemotherapy, a cystoscopy is performed to assess the response rate. If residual disease (macro- or microscopic) remains, the intent of bladder preservation is aborted, and the patient is considered for a cystectomy. On the other hand, if a complete response is achieved, then a consolidative phase of radiochemo-therapy is carried out for few more weeks, for a full course of radiochemotherapy.

The University of Erlangen has probably the largest experience in bladder preservation treatment for bladder cancer 7,48. Their multimodality experience covers a span of more than 20 years. In their initial work, rt was given alone; since 1985, chemotherapy has been given concomitantly with rt. The first chemotherapeutic agent used was carboplatin, later on replaced by cisplatin. Finally 5-fluorouracil (5fu) was added to cisplatin. A total of 415 patients with high-risk T1 (grade 3, associated carcinoma *in situ,* multiple recurrences, or tumour size greater than 5 cm) to T4 bladder cancer were treated. The treatment starts with turbt; 4 weeks later, radiochemotherapy is given. A dose of 45–54 Gy is administered to the bladder and pelvic nodes, and then the whole bladder is boosted to a dose that depends on the completeness of the initial turbt. If the procedure was complete, the bladder is boosted to 55.8 Gy; if micro- or macroscopic disease is present after turbt, the bladder is boosted to a total dose of 59.4 Gy. The daily dose is 1.8 Gy. Six to eight weeks after completion of treatment, response is assessed by cystoscopy. If the response is incomplete, cystectomy is indicated.

In 2002, Shipley *et al.* 9 published the mgh experience in bladder preservation therapy, called trimodality therapy (tmt). Clinical stages T2–T4a were included. Patients were excluded if they had pathologically proven positive nodes or hydronephrosis (an exclusion criterion that was introduced in 1993). Over 11 years, 190 patients were enrolled in five different protocols of tmt with a median follow up of 6.7 years. All mgh protocols had in common the use of an as-complete-as-possible turbt, concomitant cisplatin-based radiochemotherapy, and the recommendation of cystectomy if complete response was not achieved with the induction phase. Upon a complete response from the induction phase, a second (consolidation) phase was given to administer a curative dose of rt. The differences among the mgh protocols were the use (or not) of neoadjuvant or adjuvant chemotherapy, the addition (or not) of other chemotherapeutic agents to cisplatin during rt, and the radiation fractionation schedule. These pilot studies were the foundation on which the rtog developed several investigative protocols.

In 1993, Housset *et al.* 5 published the prospective experience in bladder preservation treatment of the Hôpital Necker in Paris. That protocol included 54 patients, 42% of whom had an abnormal intravenous pyelogram and 7% of whom had positive pelvic nodes by computed tomography scan. As in the Boston experience, the protocol consisted of turbt followed by radiochemotherapy. The drugs used were 5fu and cisplatin, and the rt was given in a bi-fractionated split course. On days 1, 3, 15, and 17, 3 Gy were administered to the pelvis on a twice-daily schedule. Chemotherapy was given on days 1, 2, 3, and 15, 16, 17. Six weeks later, a cystoscopy with deep biopsies was performed to define the next course of treatment. If residual tumour was present at cystoscopic assessment, radical surgery was then carried out. Patients achieving a complete response would continue with radiochemotherapy or could undergo a cystectomy. If the patient continued rt, a similar scheme of radiochemotherapy was given on days 64, 66, 78, and 80. The only differences in this consolidation phase were that the dose per fraction was reduced to 2.5 Gy twice daily and only the bladder volume was treated. Interestingly, Housset *et al.* 5 reported that the first 18 patients who achieved a complete response with induction radiochemotherapy and subsequently underwent a planned cystectomy evidenced a complete response (by pathology) in the bladder; moreover, all the resected pelvic nodes were also pathologically negative. Updated results of this original publication with 120 patients were presented in 1997 49.

**FIGURE 1:**
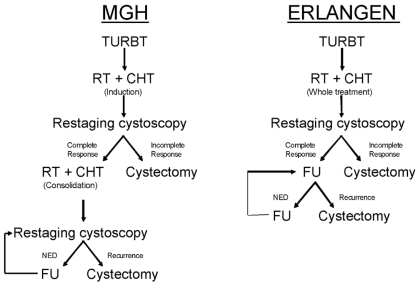
The Massachusetts General Hospital (mgh) and University of Erlangen therapeutic algorithms. turbt = transurethral resection of the bladder tumour; rt = radiation therapy; cht = chemotherapy; ned = no evidence of disease; fu = follow-up.

Despite the fact that the foregoing protocols have the same objective (bladder preservation) and present several similarities, they have distinct differences that are worth mentioning. Shipley’s group in Boston 9 and the University of Paris 5 opted for an induction treatment, after which the patient is scoped so that a decision can be made between completing a radical dose of radiochemotherapy or undergoing a cystectomy if residual disease is found. The University of Erlangen 7 policy is to give the full combined treatment up front and only then to assess cystoscopically, performing a cystectomy in the case of residual disease (Figure 1).

From a theoretical viewpoint, each approach has advantages. Giving the full treatment up front avoids a split course of radiotherapy that could reduce the chance of disease control; the Boston and Paris approaches “prioritize” the fact that, in nonresponders, surgery would not be delayed and would be carried out after a lower dose of radiation, reducing the probability of complications (preoperative dose). On the other hand, the Erlangen approach gives more time to “slow-responding” tumours to achieve a complete response, theoretically reducing the number of unnecessary cystectomies.

Another major difference between the protocols is the radiotherapy fractionation scheme used. The University of Erlangen uses a standard fractionation scheme; the University of Paris uses a bi-fractionated split course; and the Boston group has used several protocols, changing fractionation schemes over the years. Interestingly, none of the protocols has proved to be superior.

The rtog has carried out several investigative protocols exploring conservative treatment of bladder cancer. The rtog 85-12 protocol 50 was the first trial developed. It consisted of an induction phase combining rt with cisplatin (100 mg/m^2^ on days 1 and 22). Complete responders received additional rt and a third dose of cisplatin. Encouraging results of this first study led to several others. The rtog 88-02 protocol 51 was a phase ii trial that evaluated the toxicity of adding neoadjuvant methotrexate, cisplatin, and vinblastine (mcv) to the combined treatment. Treatment was well tolerated, and a randomized phase iii trial followed (rtog 89-03) to assess the efficacy of neoadjuvant mcv 52. That study was stopped early, with only 123 patients enrolled, because of slow accrual and excessive toxicity in the mcv arm, including 4 treatment-related deaths. The addition of neoadjuvant mcv did not show any benefit in terms of complete response rate, overall survival, or bladder preservation rate, and so the strategy was abandoned. The next protocol (rtog 95-06) evaluated hypofractionated radiotherapy 53. The regimen tested was the accelerated and hypofractionated scheme developed by the University of Paris 5. Overall survival was encouraging: 83% at 3 years, with 66% of patients surviving this period with an intact bladder. A 21% grade 3–4 genitourinary toxicity rate was a concern. Protocol 97-06 evaluated hyperfractionated radiotherapy (twice daily) with concomitant cisplatin (20 mg/m^2^ the first 3 days of each treatment week) followed by adjuvant mcv (3 cycles) 54. Protocol rtog 99-06 also evaluated hyperfractionated rt, but at a slightly lower daily dose. The main and novel changes in that study were the addition of paclitaxel to the concurrent cisplatin and the introduction of gemcitabine plus cisplatin in the adjuvant setting. A report in abstract form from 2005 55 shows an excellent complete response rate of 87% with 32% grade 3–4 toxicity during rt and 74% during adjuvant chemotherapy. Protocol rtog 02–33, which aims to assess the efficacy of paclitaxel compared with 5fu, both given concomitantly with cisplatin and rt, has recently closed to accrual. Finally, an ongoing study (rtog 05–24) is designed to test if the addition of trastuzumab to paclitaxel is beneficial in patients with overexpression of the human epidermal growth factor receptor (her2/*neu*). That trial is intended for non-cystectomy candidates.

To our knowledge, the only randomized trial that compared rt with rt plus chemotherapy for invasive bladder cancer was the study carried out by the National Cancer Institute of Canada 4. Their trial randomized 99 patients with muscle-invasive bladder tumours to a preoperative dose of radiotherapy with or without cisplatin. A radical cystectomy or a radiotherapy boost to the bladder was then done. This second “choice” was not randomly assigned. Pelvic progression-free survival was significantly increased in the cisplatin arm (5-year pelvic relapse-free survival: 40% vs. 59%; *p* = 0.036). This reduction in the risk of pelvic failure was not limited to patients treated with definitive rt, but was also seen in patients who underwent cystectomy. In patients who opted for conservative treatment, complete clinical response at cystoscopy after the induction phase and bladder preservation rate at last follow-up were more often seen in the combined arm. Of patients who were randomized to the combined arm and who underwent a cystectomy, 54% presented no evidence of invasive disease in the removed bladder, as compared with 40% in the rt alone arm (*p* = nonsignificant). A trend was noted for increased overall survival and decreased rate of failure of any type in the cisplatin arm, but because of the small number of patients, pelvic progression-free survival was the only variable that reached statistical significance.

## 4. DISCUSSION

### 4.1 Survival and Local Control

**FIGURE 2:**
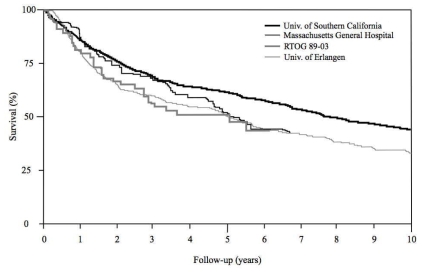
Overall survival curves from the Massachusetts General Hospital 9, the University of Erlangen 7, the Radiation Therapy Oncology Group (rtog) 89-03 52, and the University of Southern California 2 studies. Notably, in the cystectomy series from the University of Southern California, 39% of the patients had pT1 or lesser disease, which may explain the apparent better outcome as compared with bladder preservation protocols. Trimodality therapy series using various approaches yield similar 5-year overall survival rates.

Survival in modern bladder preservation series has improved over that in older publications, when chemotherapy was not given concomitantly with radiotherapy 7,9,33–35,37–39. The current 5-year overall survival ranges from 49% to 67% 7,9,50–52,56. At 10 years, overall survival is 31%–36% 7,9. Figure 2 shows overall survival curves from three bladder preservation protocols 7,47,52 and from one of the largest surgical series 2. Despite small differences in design, the conservative bladder studies show similar survival outcomes. The survival curve from the cystectomy series includes the 39% of patients with pT1 or lesser disease. If patients with only muscle-invasive disease are analyzed, the 5- and 10-year overall survival rates for cystectomy are 48% and 32% respectively— identical to the results achievable with tmt.

In Erlangen 7, the first patients were treated with rt alone; chemotherapeutic agents were then added concomitantly with radiotherapy. The complete response and the 5-year overall survival rates improved with each change (Table I). Whether these improvements are truly related to the newer approaches or to other factors such as patient selection (larger proportion of T1 tumours in the later series), stage migration, or a shorter follow-up, the effect on outcomes remains to be determined.

Approximately 12%–40% of patients will not achieve a complete response with tmt and will be considered for an immediate cystectomy 5,7,9,48–51,53–56. Among the complete responders, approximately one third (14%–50%) will fail locally. Approximately 60% of local failures are superficial; the remaining 40% are muscle-invasive 5,7,9,51–53. Most of the superficial recurrences are managed in a conservative way (see the “Recurrences After Bladder Preservation” subsection). Overall, the 5-year survival rate with an intact bladder ranges from 38% to 51%, representing a rate of approximately 80% bladder preservation in long-term survivors 7,9,51–56.

Table II summarizes the characteristics and results of various series. The highest complete response rates are from the two series that combined cisplatin with another chemotherapeutic agent concomitantly with radiotherapy. Both [the University of Erlangen 7,48, combining cisplatin with 5fu (Table I), and rtog 99-06 55, combining cisplatin plus paclitaxel (Table II)] report a similar complete response rate of 87%. These two series have included more patients with early disease than other series have (90% of T2 tumours in the rtog 99-06 55 study, and 48% T1 in the Erlangen series 48), and this cohort composition could explain, in part, the reason for the higher complete response rates. Notably, other series using multidrug regimens combined with hypofractionated rt did not achieve the same complete response rates 5,49,53.

### 3.5 Prognostic Factors

Several variables have been correlated with survival and bladder preservation rates in organ-preservation treatments for bladder cancer. Tumour stage, as expected, affects local control and survival 3,7,9,50,56. The complete response rate for T2 tumours ranges from 71% to 88% and for T3–4 tumours, from 57% to 72% 7,9,50,56. Recently, Weiss *et al.* 48 published a series of 112 patients treated with rt and concomitant cisplatin/5fu, reporting freedom from local relapse at 5 years of 66% for T1 and 51% for T2–4. In the Boston experience 9, the 5-year overall survival for T2 tumours was 62%, and for T3–4 tumours, it was 47%. The 5-year disease-specific survival was 74% for T2 and 53% for T3–4. The Erlangen series 7, with 415 patients, reported 5- and 10-year overall survival rates of 75% and 51% respectively for T1 tumours. Those rates decreased to 56% and 32%, 44% and 26%, and 17% and 9% for T2, T3, and T4 tumours respectively. In the multi-institutional rtog protocol 85-12 50, a 4-year overall survival of 64% for T2 and 24% for T3–4 tumours was observed. In an unselected group of patients, Chung *et al.* 3 reported overall survival for T2 tumours of 43% and 27% at 5 and 10 years respectively. These same figures for T3–4 disease dropped to 16% and 7% respectively (*p* < 0.01). In our opinion, the two major reasons for the poor outcomes in this last series were the unselected nature of the patients assigned to treatment (more than 25% were not medically fit for surgery) and, most importantly, the 73% of patients treated without concomitant chemotherapy. This same study also showed that, in a subset of T2N0M0 patients, the local relapse-free rate was significantly higher if the tumour was 2 cm or smaller, indicating that not only T stage, but also the actual size of the tumour are important predictive factors affecting the rate of disease recurrence.

The presence of hydronephrosis at diagnosis has been shown by some studies to significantly affect complete response rates. According to the Boston experience 9, in the presence of hydronephrosis, the complete response rate was 37% as compared with 68% when hydronephrosis was absent. This observation led to the incorporation of hydronephrosis as an exclusion criterion in their subsequent bladder preservation protocols. Other studies 36,52 also found a significant relationship between a poor complete response rate and the presence of ureteral obstruction. Hydronephrosis has also been associated with a significantly increased risk of distant metastasis 51.

Rodel *et al.* 7 found a statistically significant association between the completeness of the turbt and both higher complete response rate and improved overall survival. The complete response rate in patients with a complete turbt was 90%, decreasing to 77% in patients with microscopic residual disease after turbt and to 54% if macroscopic residual disease was present. The 10-year overall survival rates in R0, R1, and R2 were 50%, 33%, and 18% respectively. In a series of 122 patients, Perdona *et al.* 56 also observed the same detrimental effect of an incomplete turbt. However, in other studies, the completeness of the turbt did not significantly affect the complete response rate or survival. Interestingly, however, those studies did find that the survival rate with an intact bladder was higher in patients with a complete, as compared with an incomplete, turbt (rtog 89-03: 38% vs. 64%; *p* = 0.06; Boston: 29% vs. 50%; *p* < 0.01) 52,57.

The presence of carcinoma *in situ* (cis) associated with invasive disease in the bladder is another variable that has been associated with decreased local control rates. The Princess Margaret Hospital experience 3 shows a significant increase in local relapse rates in patients with associated cis. The local relapse-free rate was 61% if cis was absent against 29% if present. The 5-year overall and cause-specific survival rates were also decreased in the presence of cis. On the other hand, other series 7,56 did not find a significant relationship between complete response rate or survival and the presence of cis. In a consensus meeting convened by the Société Internationale d’Urologie 58, the radiotherapy panel indicated that the presence of extensive cis in association with muscle-invasive bladder cancer increases the risk of a superficial recurrence after tmt, and that these patients have an increased risk of requiring a cystectomy for a future recurrence. Considering that the overall survival rate in patients that require a salvage cystectomy is similar to that in patients that never recur, the panel concluded that the presence of cis is not an absolute contraindication to the use of tmt.

The completeness of response to induction treatment is a predictive factor for metastasis, suggesting that non-responding tumours are intrinsically more aggressive and may metastasize earlier. Rodel *et al.* 7 reported that freedom from distant metastasis in complete responders was 79% and 70% at 5 and 10 years, significantly higher than in non-complete responders (52% and 48% respectively). Housset *et al.* 49 described an overall survival of 73% at 5 years in complete responders and 29% in non-complete responders (*p* < 0.0001). The same study also showed that, after achieving a complete response to induction radiochemotherapy, patients experienced no difference in overall survival at 5 years whether operated on or having completed the bladder preservation approach, suggesting that the induction radiochemo-therapy phase is an effective and selective method for optimally defining patients that are good candidates for ultimately preserving their bladder.

### 4.2 Recurrences After Bladder Preservation

The risk of developing a local recurrence after achievement of a complete response ranges from 14% to 43% at 5 years with 30%–50% of patients presenting a component of invasive disease 5,7,9,49,52. Most local recurrences appear within the first 12–24 months from the end of therapy, and thus patients need to be followed closely with repeated cystoscopies during this period 3,5,50,51,59,60. However, follow-up should not be limited to the first 2 years after treatment, because some series 3,59–61 report local recurrences as late as 5–10 years after treatment. Some of these patients can still be salvaged surgically.

Cystectomy is the treatment of choice for muscle- invasive local recurrences, and approximately 30%–40% of locally relapsing patients ultimately undergo a cystectomy 7,51,53. In the largest published series to date, salvage cystectomy for an invasive recurrence after a complete response from tmt yields 5- and 10-year disease-specific survival rates of 50% and 45% respectively 7. A non-complete response to tmt appears to be associated with a poorer prognosis, with only 21% of patients achieving 5-year disease-specific survival. One other large study 9 showed a similar disease-specific survival rate in patients who underwent a cystectomy for an incomplete response as compared with patients who were operated for a local muscle-invasive recurrence after a complete response. Whether the difference between series results from biologic tumour behaviour or from therapeutic strategy remains to be determined.

One important issue related to recurrent disease is how to treat superficial recurrences. More than 87% of patients 50–53,59,60 are typically managed with a conservative approach with turbt, with or without intravesical treatment. Those remaining are treated mostly using cystectomy. The rate of a second local recurrence in conservatively treated patients ranges from 36% 60 to 52% 59, and the risk of developing invasive disease after being treated conservatively for a superficial recurrence ranges from 0% to 19% 5,50,52,53,59,60. Those results are comparable to those obtained in *de novo* superficial tumours treated in non-irradiated bladders 62. Overall, 0% to 31% of the patients will eventually need a cystectomy after developing a superficial recurrence 50–53,59,60.

In the only two studies that analyzed in detail the management of superficial recurrences, the development of a superficial recurrence did not affect long-term disease-specific or overall survival as compared with survival in patients that did not recur. However, in both studies, long-term survival with a preserved bladder decreased by approximately 40% 59,60. In other words, superficial recurrence after tmt appears to be a negative prognostic factor for bladder preservation, but not for survival.

### 4.3 Cystectomy Rate

When a bladder preservation approach is being considered, the patient has to be aware that approximately one third of patients will not achieve a complete response. An immediate cystectomy offers the best chance of cure if the disease is still localized. Even after a complete response, compliance with frequent follow-up cystoscopies is of paramount importance, because in the case of a local recurrence, a cystectomy may be necessary. Overall, 20%–40% of patients undergo a cystectomy at some point because of incomplete response, local recurrence, or treatment complications. The probability of requiring removal of the bladder because of complications is 0%–2% 3,5,7,9,50,51,53,56.

One concern on which many urologists base their opposition to bladder preservation therapy is the potential difficulty of performing a continent urinary diversion after radical doses of radiation. In 1998, the University of Southern California published its experience in cystectomy with neobladder reconstruction in patients that had received rt to the pelvis 63. In their small series of 18 cases, the complication and continence rates were similar to those obtained in non-irradiated patients. Similar findings were presented in another series 64 of 37 women with a history of prior pelvic irradiation who underwent a pelvic exenteration for pelvic tumour recurrence or urinary diversion for vesicovaginal fistula and had an ileocecal continent urinary reservoir. In the Boston series 9, only 2 of 66 cystectomized patients (3%) underwent a continent urinary diversion; other smaller series reported rates of 21% (4/19) and even 100% (3/3) 5,6.

### 4.4 Which Drug to Use?

The largest experience in radiochemotherapy-based organ-preservation treatments for bladder cancer is with cisplatin 4,5,7,9,49,56. The University of Erlangen studies 7 evolved by changing only the chemotherapeutic agent, keeping unchanged the rest of the treatment components. This “simple and ordered” step in the evolution of the protocol made it easier to evaluate the effect of various drugs in the outcome 65. Over the years, the investigators moved from rt alone to chemo-rt using a single agent, and later, to combined chemotherapy agents. The complete response rate was significantly enhanced by the combined approach, translating into improved survival rates (Table I). Whether the addition of 5fu truly improves results still needs to be confirmed. The newer regimens included a larger number of patients with earlier disease, and part of the improved outcome may be related purely to that factor.

Similar complete response rates to multidrug concomitant regimens were obtained with cisplatin– paclitaxel in rtog protocol 99-06 55. In contrast to the German experience, the rtog trial used hyperfractionated rt and also added cisplatin and gemcitabine in the adjuvant setting. The final results are not yet available, but an interim analysis 55 revealed that 87% of patients completed the treatment per protocol and that the estimated 2-year survival with an intact bladder is 69%.

Currently, no solid data support one chemotherapy agent over the other. Cisplatin is the agent most frequently used, and we believe that it is the one that should be considered for treating patients outside the setting of an investigative protocol. Based on the large experience of the University of Erlangen with 5fu plus cisplatin 7, that combination can also be considered for selected patients.

### 4.5 Quality of Life

To be considered an alternative therapy with respect to surgery, a bladder preservation treatment has to preserve a native bladder capable of performing its function in a normal, or almost normal, way. A review article by Wright and Porter 66 shows the difficulty in assessing quality of life (qol) in bladder cancer patients. The most disturbing symptom is probably incontinence. Surgical series report daytime continence rates of 70%–95%. Nighttime continence is more problematic, with rates of 65%–85% 28.

On the other end of the spectrum, a French National Survey 67 study reported nighttime continence rates of only 17.4% in patients with neobladder and interference with sleep in 32% of all patients. In that survey, 40% of the patients had to wear at least one pad daily. However, 90%–95% of the patients were satisfied with their urinary diversion 67,68. This discordance between continence and satisfaction is probably a result of patients interpreting and eventually becoming used to their symptoms as the price to pay to be cured of cancer.

Zietman *et al.* 69 performed urodynamic tests and a qol study on long-term survivors treated with tmt for infiltrative bladder cancer. Of the 71 patients alive at a median follow-up of 6.3 years, 68% completed a qol questionnaire, and 45% were tested with urodynamic studies. Of these patients, 75% were considered to have bladders with normal function. Five women, representing 11% of the patients, had to wear pads. Weiss *et al.* 48 analyzed 71 patients with a qol questionnaire, and 78.8% were “delighted” or “pleased” in terms of urinary function.

The risk of having to remove a bladder because of post-radiotherapy contraction is 0%–2% 9,65. The University of Erlangen 48,65 reported that only 2% of patients required a “salvage cystectomy” for “contracted bladder” (grade 4) and that 9% developed a reduced bladder capacity (grade 3) without the need for surgery, despite the fact that many patients under-went multiple turbt procedures before tmt.

Zietman *et al.* 69 demonstrated that 54% of men had erections hard enough for intercourse and that 59% were satisfied with their sex life after conservative treatment. These rates compare favourably with a contemporary questionnaire-based study that reported an 87% rate of inability to maintain an erection after radical surgery 70. Following nervesparing cystoprostatectomy, potency rates of 42% have been reported 71.

## 5. FUTURE DIRECTIONS

The current experience with tmt protocols are with conventional or three-dimensional (3-D) conformal rt. Intensity-modulated rt (imrt) and image-guided rt allow for highly conformal dose delivery to target volumes, with decreased dosage to normal organs at risk. Use of these techniques in bladder cancer patients may lead to further improvements in local control rates without increasing the risk of side effects. The superiority of these techniques over 3D-conformal rt with respect to tumour control and side effects has been shown both dosimetrically and clinically in many studies. The use of imrt in bladder cancer is still preliminary, and further experience is obviously needed. However, the prospect of delivering a higher biologically equivalent dose (concomitant boost technique) or of dose escalation under conventional fractionation is an attractive alternative that is now being explored in prospective trials.

Although platinum and taxane compounds are now the most frequently used chemotherapy agents in bladder preservation studies, new promising data emerging from the use of gemcitabine are making that agent another potential drug for use in tmt. Kent *et al.* 6 from the University of Michigan reported on a phase i trial using twice-weekly gemcitabine (27 mg/m^2^) concomitantly with conventionally fractionated rt. The toxicity was acceptable, and 65% of the patients were disease-free with a native bladder at a median follow-up of 43 months. In a similar phase i study with weekly gemcitabine and concomitant hypofractionated rt, 7 of the 8 patients were disease-free at a median follow-up of 19.5 months 8. These preliminary but encouraging results will now be tested in a randomized phase ii study by the rtog. Interestingly, the rtog will, for the first time in bladder cancer, allow the use of imrt for delivery of the rt component. More than 50% of patients with invasive bladder cancer die from metastatic disease, and therefore more effective adjuvant systemic therapy is required to truly improve overall survival in these patients.

Molecular profiling is an exciting area that holds promise that clinical outcomes might be improved by identifying the mechanisms and targets associated with the growth of a tumour and by developing potential and specific therapeutic agents. A significant number of laboratory and clinical investigations have uncovered molecular markers in bladder cancer, and some of these are being actively investigated.

A recent rtog report 72 analyzing histopathologic material from four bladder preservation trials (rtog 88-02, 89-03, 95-06, and 97-06) described a significant correlation between her2/*neu* overexpression and decreased complete response rates. That finding led the rtog to develop an ongoing trial (rtog 05-24) in which patients with her2/*neu* overexpression receive trastuzumab, a monoclonal antibody against her2/*neu,* concomitantly with paclitaxel and rt.

The data concerning overexpression of the epidermal growth factor receptor (egfr) is conflicting. In surgical series, egfr has been adversely associated with prognosis 73, but in tmt series from the rtog, egfr expression appeared to correlate significantly with improved outcome 72.

Further work for bladder cancer patients is clearly needed in this exciting new molecular arena. Numerous trials are currently ongoing, and hopefully this revolution in molecular and cellular biology will lead to higher cure rates.

## 6. CONCLUSIONS

Bladder preservation therapy with tmt is a safe treatment that achieves survival rates similar to those achieved in modern cystectomy series; it should be considered an alternative first-line treatment for invasive bladder cancer in selected patients. The 5- and 10-year survival rates for muscle-invasive tumours are approximately 50% and 35%, comparable to the 48% and 32% achievable with cystectomy 2. Approximately 80% of long-term survivors will preserve a native bladder, and approximately 75% of them will have a normal-functioning bladder.

The ideal candidate for bladder preservation therapy is a patient with a small tumour, stage T2, in whom a complete turbt is achievable, who has no associated cis or hydronephrosis, and who is medically fit to receive chemotherapy.

When a bladder preservation treatment is considered, the patient has to fully understand that there is a 1 in 3 chance of their requiring cystectomy at some point during treatment or follow-up, and that the survival rates described take into account cystectomy-salvaged patients. Salvage is the reason that frequent follow-up with cystoscopy is a major component of any bladder preservation protocol.

Finally, the multidisciplinary cooperation of the urologist, the radiation oncologist, and the medical oncologist is of major importance—not only to deliver treatment in a timely manner, but also to actively follow the patient and to provide the earliest possible salvage treatment if needed.

## Figures and Tables

**TABLE I:** The 5-year overall survival and complete response rates from the University of Erlangen based on changes in therapeutic approaches over the years 7,48

Treatment	Overall survival (%)	Complete response (%)
rt alone	40	61
rt+carboplatin	45	66
rt+cisplatin	62	82
rt+cisplatin+5fu	65	87

rt = radiation therapy; 5fu = 5-fluorouacil.

**TABLE II:** Results of trimodality therapy in bladder preservation studies

Study	Treatment	Pts (*n*)	Median follow-up (months)	cr (%)	Overall survival (%)			Bladder preservation in long-term survivors (%)	lr rate (%)
					5-year	10-year	5-year with bladder preservation		
rtog 88-02 51	Neoadjuvant mcv plus c–rt	91	46	75	62 (4-year)		44 (4-year)	60	50
rtog 89-03 52	With/without neoadjuvant mcv plus c–rt	123	61	59	49		38	78	43
rtog 95-06 52	c/5fu/hypofx bid split-course rt	34	29	67	83 (3-year)		66 (3-year)	79	45
rtog 97-06 54	c/hyperfx bid rt with adjuvant mcv	47	26	74	61 (3-year)		48 (3-year)	79 (3-year)	19
rtog 99-06 55	c/p/hyperfx bid rt with adjuvant g/c	47	30	87	79 (2-year)		69 (2-year)	87 (2-year)	18
University of Paris 5	c/5fu/hypofx bid split-course rt	120	49	77	63				17
mgh^9^	Varying protocols (see text)	190	80	64	54	36	45	83	40
University of Erlangen 7	Varying protocols (see Table I)	415	60	72	51	31	42	82	35

Pts = patients; cr = complete response; lr = local recurrence; rtog = Radiation Therapy Oncology Group; mcv = methotrexate, cisplatin, vinblastine; c = cisplatin; rt = radiation therapy; 5fu =5-fluourouracil; hypofx = hypofractionated; bid = twice daily; hyperfx = hyperfractionated; p = paclitaxel; g = gemcitabine; mgh = Massachusetts General Hospital.
